# Benchmarking the Wilmer general eye services clinics: baseline metrics for surgical and outpatient clinic volume in an educational environment

**DOI:** 10.1186/s12909-016-0556-x

**Published:** 2016-01-27

**Authors:** Eric Singman, Divya Srikumaran, Kathy Hackett, Brian Kaplan, Albert Jun, Derek Preece, Pradeep Ramulu

**Affiliations:** Division Chief, Wilmer General Eye Services at Johns Hopkins Hospital, Baltimore, USA; Residency Program Director, Wilmer Eye Institute at Johns Hopkins Hospital, Baltimore, USA; Project Manager, Wilmer Eye Institute Information Technology, Baltimore, USA; Financial Analyst, Wilmer Eye Institute, Baltimore, USA; Vice Chair for Education, Wilmer Eye Institute, Baltimore, USA; Principal and Executive Consultant, BSM Consulting, Baltimore, USA; Division Education Champion for Glaucoma, Wilmer Eye Institute, Baltimore, USA

**Keywords:** electronic surgical posting, benchmark, resident education, surgical volume, patient volume, practice management

## Abstract

**Background:**

The Wilmer General Eye Services (GES) at the Johns Hopkins Hospital is the clinic where residents provide supervised comprehensive medical and surgical care to ophthalmology patients. The clinic schedule and supervision structure allows for a progressive increase in trainee responsibility, with graduated autonomy and longitudinal continuity of care over the three years of ophthalmology residency training. This study sought to determine the number of cases the GES contributes to the resident surgical experiences. In addition, it was intended to create benchmarks for patient volumes, cataract surgery yield and room utilization as part of an educational initiative to introduce residents to metrics important for practice management.

**Methods:**

The electronic surgical posting system database was explored to determine the numbers of cases scheduled for patients seen by residents in the GES. In addition, aggregated residents’ self-reported Accreditation Council for Graduate Medical Education (ACGME) surgical logs were collected for comparison. Finally transactional databases were queried to determine clinic volumes of new and established patients. The proportion of resident surgeries (1^st^ surgeon and assistant) provided by GES patients, cataract surgery yield and new patient rates were calculated. Data was collected from July 1^st^, 2014 until March 31^st^, 2015 for all 16 residents (6 third year, 5 second year and 5 first year).

**Results:**

The percentage of cataract, oculoplastics, cornea and glaucoma surgeries in which a resident was 1^st^ surgeon and the patient came from the GES was 91.3, 76.1, 65.6, and 93.9 respectively. The new patient rate was 28.1 % and room utilization was 50.4 %. Cataract surgery yield was 29.2

**Discussion:**

The GES provides a significant proportion of primary surgeon opportunities for the residents, and in some instances, the majority of cases. Compared to benchmarks available for private practices, the new patient rate is high while the cataract surgery yield is low. The room utilization is lower than the 85 % preferred by the hospital system. These are the first benchmarks of this type for an academic resident ophthalmology practice in the United States.

**Conclusions:**

Our study suggests that resident-hosted clinics can provide the majority of surgical opportunities for ophthalmology trainees, particulary with regard to cataract cases. However, because our study is the first academic resident practice to publish metrics of the type used in private practices, it is impossible to determine where our clinic stands compared to other training programs. Therefore, the authors strongly encourage ophthalmology training programs to explore and publish practice metrics. This will permit the creation of a benchmarking program that could be used to quantify efforts at enhancing ophthalmic resident education.

## Background

The Wilmer General Eye Services (GES) at the Johns Hopkins Hospital (JHH) is the clinic where residents provide supervised care to comprehensive ophthalmology patients; the residents are encouraged to work with a significant degree of autonomy in this setting. Although the GES has been in service since 1925, it has been an official division of Wilmer since 2011. At that time, the GES was provided with an administrative team headed by a full time medical director and clinic manager whose responsibilities included identifying, tracking and optimizing the divisional educational contribution to resident training. In addition to these duties, the GES administration was tasked with working closely with the Wilmer residency program director and Wilmer Vice Chair for Medical Education to enhance the training experience in the GES. Toward this end, the GES Division Chief co-directed a program called the Resident Education Task Force (RETF), a Wilmer-wide effort initiated by the chairman to evaluate resident education. The RETF identified strengths to enhance and weaknesses to mitigate or eliminate, and then developed a system whereby the processes for improvement are continually monitored, evaluated and adjusted using specific metrics such as resident scores on the Ophthalmology Knowledge Assessment Profile (OKAP), surgical numbers for residents, numbers of publications and poster presentations, and resident satisfaction scores such as those mandated by the Accreditation Council for Graduate Medical Education (ACGME). One of the changes implemented by the RETF was the creation of a Division Education Champion (DEC), a faculty member from each division who became responsible for clarifying, disseminating, implementing and monitoring the core competencies of that division, enabled by protected time and salary support. This approach had been suggested by the American Academy of Ophthalmology and the American Board of Ophthalmology [1]. The residency education committee was then reformulated as the Program Evaluation Committee, consisting of the DECs as well as resident volunteers from each post-graduate year. The DECs were also instrumental in directly improving the clinical experience in the GES by facilitating the addition of specialty clinics running concurrently and co-located with the residents’ own comprehensive clinics. In academic year 2014–15, there were GES clinics devoted to Oculoplastics, Glaucoma, Anterior Segment/Cornea, Retina (laser, surgery and injections), Neuro-ophthalmology (specializing in traumatic brain injury), Pediatrics, Genetics and Optometry. In academic year 2015–16, a clinic in uveitis will be added. The goal of these enhancements is to create a department-within-a-division model, ensuring that residents capture a wide variety of clinical and surgical opportunities by referring them internally within the GES. Residents currently host their comprehensive continuity clinic in the GES one day each week and then rotate in the GES specialty clinics or in attending-hosted clinics throughout the hospital system. There are opportunities to identify surgical cases in all of these venues.

The DEC representing the GES division’s portfolio includes a mandate to demonstrate the educational impact of a clinic where residents are the chief providers of continuity care. In addition, the GES DEC is tasked to instruct residents in the profession of medicine, including but not limited to coding, compliance, good charting habits, issues related to patient satisfaction, delegation of care, understanding scope of practice, interactions with colleagues across divisions and departments, practice management and ethics; these are all priorities described by the American Academy of Ophthalmology [2].

Identifying a Wilmer Eye Institute faculty member to oversee the training of professionalism to residents may be a unique approach, but it is aligned with the core competencies co-developed by the American Board of Medical Specialties (ABMS) and the Accreditation Council for Graduate Medical Education (ACGME) [3]. Indeed, a recent survey of ophthalmology residents themselves has indicated that training in aspects of professionalism, such as practice management, could be enhanced [4]. Teaching professionalism to residents implies that they will develop an understanding of some of the business processes and metrics involved in practicing ophthalmology. The increasing complexity of the business environment and decreasing latitude in which healthcare dollars are spent add priority to this task. Therefore it seems reasonable to support the educational mission of an academic outpatient ophthalmology clinic by identifying, understanding, managing and teaching metrics such as clinic and surgical volumes. Although these metrics are important from a financial perspective, they take on additional academic importance, since they can be used to introduce residents to the process of practice management. This study, as part of an ongoing effort to document the evolution of resident surgical education at the Wilmer Eye Institute, sought to establish key benchmarks that would be important to any academic ophthalmology outpatient clinic hosted by residents. To the authors’ knowledge, a study of this type has not been published for a United States academic outpatient ophthalmology clinic.

## Methods

Data for this study were collected for the period between July 1, 2014 and March 31, 2015 for all 16 residents (6 third year, 5 second year and 5 first year). Representative surgical cases generated by patients visiting the Wilmer General Eye Services (GES) Clinic were gleaned from the surgical scheduling database by tracking Current Procedural Terminology (CPT^TM^) and Operating Room Management Information System (ORMIS^TM^) codes for patients whose procedures were scheduled by a modified version of the electronic surgical posting (e-posting) sheet. This modification permitted the physician scheduling a surgery to indicate whether the patient emanated from the GES. The codes tracked by the e-posting system are presented in Table 1. Although the case-lists extracted from the e-posting system contained patient names and medical record numbers, they were visible only to the faculty members overseeing the resident providers. In addition, resident surgical cases were gleaned from self-reported resident ACGME surgical logs. These logs contain no personal health information and were aggregated for the entire cohort of residents so that no individual resident’s data was available. Using these methods we were able to identify virtually all surgical cases generated by the GES for residents during the time period noted.

**Table 1:** Representative Surgical Cases Tracked in e-posting for GES Patients

Codes format: *CPT*/**ORMIS**
**Cornea/Anterior Segment**:
*66984* **/9574:** extracapsular cataract extraction with intraocular lens implantation
*65710*/**9480:** keratoplasty lamellar
*65730* **/9585:** keratoplasty (penetrating, phakic)
*65750* **/9445:** keratoplasty (aphakia)
*65755* **/9446:** keratoplasty (pseudophakia)
*65756/* **1242:** keratoplasty (endothelial)
*65770* **/9377:** keratoprosthesis
*65780* **/1096:** amniotic membrane transplant
*65426* **/9438:** excision pterygium with graft
**Glaucoma**:
*66170*/**9349:** trabeculectomy ab externo in absence of previous surgery
*66172*/**9349:** trabeculectomy ab externo with scarring from previous ocular surgery or trauma
*66180* **/9484:** aqueous shunt to external reservoir
*66710* **/9566:** Ciliary body destruction; cyclophotocoagulation
*66250* **/9590:** revision or repair of operative wound of anterior segment, any type, early or late, major or minor procedure
**Oculoplastics**
*15823* **/4478:** blepharoplasty
*21390* **/5842:** Open therapy of orbital floor blowout fracture, transantral periorbital approach with implant
*65105* **/8998:** enucleation w/implant, muscles attached
*67840* **/9514:** excision of lesion of eyelid (except chalazion) w/o closure or w/ simple direct closure
*67904* **/9437:** external approach blepharoptosis repair, levator resection,
*67916* **/9539:** repair of ectropion, excision tarsal wedge
*67917* **/9539:** extensive tarsal strip operation
*67924* **/9538:** tarsal strip or capsulopalpebral fascia repairs operation
*67930* **/9515:** Suture of recent wound, eyelid, involving lid margin, tarsus, and/or palpebral conjunctiva direct closure; partial thickness
*67935* **/9515:** Suture of recent wound, eyelid, involving lid margin, tarsus, and/or palpebral conjunctiva direct closure; full thickness
*68720* **/9542:** Dacryocystorhinostomy (fistulization of lacrimal sac to nasal cavity)
**Pediatrics/Strabismus**:
*67311*–*67335* inclusive**/8986:**
i.* 67311* one horizontal muscle
ii.* 67312* two horizontal muscles
iii.* 67314* one vertical muscle except superior oblique
iv.* 67316* two or more vertical muscles except superior oblique
v.* 67318* superior oblique
*68815* **/9347:** probing of nasolacrimal duct, with or without irrigation; with insertion of tube or stent
*68816* **/9347:** Probing of the nasolacrimal duct, with or without irrigation, with transluminal balloon catheter dilation
*66850* **/9573:** Removal of lens material; phacofragmentation technique
*67010* **/9353:** subtotal removal of vitreous with mechanical vitrectomy
**Retina**:
*67113*/**9353:** Repair of complex retinal detachment (eg, proliferative vitreoretinopathy, stage C-1 or greater, diabetic traction retinal detachment, retinopathy of prematurity, retinal tear of greater than 90 degrees), with vitrectomy and membrane peeling, may include air, gas, or silicone oil and/or removal of lens
*67108*/**9353:** Repair of retinal detachment; with vitrectomy, any method, with or without air or gas tamponade, focal endolaser photocoagulation, cryotherapy, drainage of subretinal fluid, scleral buckling, and/or removal of lens by same technique
*67042*/**9353:** Vitrectomy, mechanical, pars plana approach with removal of internal limiting membrane of retina (eg, for repair of macular hole, diabetic macular edema), includes, if performed, intraocular tamponade (i.e., air, gas, silicone oil)
*67107*/**9353:** Repair of retinal detachment; scleral buckling
*67040*/**9353:** Vitrectomy, mechanical, pars plana approach; with endolaser panretinal photocoagulation
*67041*/**9353:** Vitrectomy, mechanical, pars plana approach; with removal of preretinal cellular membrane
*67039*/**9353:** Vitrectomy, mechanical, pars plana approach; with focal endolaser photocoagulation
*67036*/**9353:** Vitrectomy, mechanical, pars plana approach
**Uveitis**:
*66982* **/9360:** complex cataract requiring devices or techniques not generally used in routine cataract surgery (eg., iris expansion device, suture support for IOL, primary posterior capsulorrhexis) or performed in patients in the amblyogenic developmental stage
*67027* **/9364:** implantation of intravitreal drug delivery system (Retisert^TM^, Vitrasert^TM^, Ozurdex^TM^)

Clinic volumes were tracked using transactional analysis of hospital billing records. Benchmarks we analyzed include the contribution of GES patients to overall resident surgical volumes, monthly and aggregate volumes of representative surgeries from the various ophthalmic subspecialties, proportion of new and established patient visits, proportion of cataract surgeries to new and total patient visits, and room utilization.

Room utilization was calculated based upon a hospital-wide formula which assumes that there are 251 clinic days/year, the work day is 8 hours long, and maximum utilization is 1 patient/hour. The target for utilization is 85 % at a maximum; the formula for utilization rate is: 100 x Va/Vm, where Va is the number of patient visits for the year and Vm is 85 % of the maximum number of patients that can be seen in the clinic. Since the GES has 17 examination rooms (each hosting only one patient visit at any time), the maximum number of patients that can be seen according to the hospital utilization formula is 17(rooms (i.e., patients)) x 251 (days/year) x 8 (hours/day) = 34,136; 85 % of that is 29,016 patients.

This study has been approved by the JHH Institutional Review Board (IRB) as non-human subjects research; as such, the need for consent was waived.

## Results

Table 2 provides the numbers of surgeries tracked by the GES through the e-posting system (column 1) as well as the numbers of surgeries logged by the residents themselves for cases during which the resident was the primary surgeon (column2). Notably, the ACGME-resident logs are aggregated for the entire program and the case numbers are distributed across the corresponding CPT codes. Surgical subcategories that the DEC’s explored included procedures involving the cornea/anterior segment, glaucoma, oculoplastics/eyelid, pediatrics/strabismus, retina/vitreous, uveitis and cataract/intraocular lens. The third column of the table shows the numbers of the resident logged-cases filtered through those codes tracked by the GES. Many of the codes the DEC’s chose not to track were for procedures that would be expected to be performed on patients coming through the emergency department rather than the outpatient clinic, particularly those related to trauma, including intra-ocular foreign bodies, ruptured globes, eyelid lacerations and orbital fractures. Column 4 shows the percentage of cases emanating from GES in which the resident was first surgeon (value in column 1 divided by value in column 3). The highlighted results are those classes of surgeries where the GES contributed 2/3 or more of the total resident surgical caseload. These include cornea/anterior segment, glaucoma, oculoplastics, uveitis and cataract/lens. The GES contributed more than 1/3 of the pediatrics/strabismus cases and nearly ½ of the retina/vitreous cases.

**Table 2:** Numbers of Surgeries

Division	GES cases in e-posting^a^	Resident logged as 1^st^ surgeon, ACGME codes^b^	Resident logged as 1^st^ surgeon, GES codes^c^	Percentage of Resident Cases from GES (1^st^ surgeon)^d^
Cornea/Anterior Segment	46	85	49	93.9
Glaucoma	86	209	113	76.1
Oculoplastics/Eyelid	120	388	183	65.6
Pediatrics/Strabismus	56	157	148	37.8
Retina/Vitreous	217	460	448	48.4
Uveitis	1	0	0	100
Cataract/Lens	501	579	549	91.3

^a^All GES cases registered in e-posting associated with the tracked codes noted in Table 1

^b^All resident-logged cases in ACGME-mandated logs; ACGME does not limit codes that can be entered

^c^Any resident-logged cases from ACGME-mandated logs filtered through the set of codes tracked by the GES (i.e., the codes in Table 1)

^d^ This percentage is the number of cases emanating from the GES (column 1) divided by the total number of surgeries logged by the resident when the resident was primary surgeon (column 3)

During the study period, the numbers of new and established patient visits were 4114 and 10,512, respectively, and the ratio of new to total (14,626) patients was 28.1 %. The ratios of GES-surgical cases to total patients, GES-cataract cases to new patients, and GES-cataract cases to total patients was 9.7 %, 12.2 %, and 5.8 % respectively. Room utilization, based upon the JHH standard was 50.4 %. Finally, the no-show rate for the GES outpatient clinic averaged 22.1 % for the study period and the same-day cancellation for the operating room used by our patients averaged 4 % during the study period.

## Discussion

The key finding of this study is that the GES, a clinic where residents serve as primary providers for patients (with appropriate supervisory safeguards), provides residents with a substantial number of high quality (i.e., first surgeon as opposed to assistant) surgical opportunities in most of the major ophthalmic disciplines. Recognizing that residents host their continuity clinic in the GES only one day each week, it seems reasonable to suggest that a resident clinic can be a viable source of significant surgical volumes and therefore a worthwhile model for surgical training. At present there are no other benchmarks to compare these data, and it may be useful to survey academic training sites to determine whether they host a clinic similar to the GES and if so, to query how their experiences compare.

Benchmarking, i.e., comparing a specific measure to a standard or average, has received great emphasis by the American Academy of Ophthalmology and it is now possible for a private practice to compare metrics such as financial performance and practice efficiency [5]. An international benchmarking initiative for eye hospitals was launched in 2001 and a report evaluating its progress was published in 2010 [6]. While the stakeholder hospitals showed very limited success in achieving the key conditions for benchmarking explored in the study, the exercise of benchmarking itself stimulated a more positive environment for learning and interactions on tactical and operational levels. Therefore, it must be mentioned that the benchmarks reported in our present study are simply unavailable for academic faculty or resident ophthalmology practices. Fortunately, some of these metrics are readily available for private practices. For example, two commonly employed measures of productivity are cataract surgery yield (total patient encounters/cataract surgeries performed) and new patient ratio (number of new patients/number of total patients). In our practice, these metrics were 29.2 and 28.1 %, respectively. A recent publication on benchmarking suggests that our cataract surgery yield would be considered in the bottom 25^th^ percentile compared to a private practice (i.e., below the “healthy range”) while our new patient ratio would be considered near the top 25^th^ percentile (i.e., high performing within the “healthy range”) [7]. It may be that the “healthy range” for cataract surgeries will be found to be lower for academic practices, since a practicing ophthalmologist would presumably be more comfortable performing cataract surgeries on patients with less severe vision impairment than might a third year ophthalmology resident. Furthermore, unlike most comprehensive ophthalmology practices, the GES treats even the youngest of pediatric patients. Since these patients would not be expected to require cataract surgery often, their presence would lower the cataract surgery yield based upon the formula cited. Finally, it is not possible to perform the same amount of surgeries in an educational setting because the time needed for each case is expected to be longer when residents are involved and so fewer cases must be scheduled for each day in the operating room [8–11].

The room utilization rate for academic practices in US medical schools in 1996 (not specific to ophthalmology) was reported as being “40 to 50 %” [12]; our utilization rate (50.4 %) falls within this range and is lower than preferred (i.e., 85 %) by JHH. The room utilization for outpatient clinics of other specialties at JHH is provided in Table 3. Notably, the GES is the only outpatient clinic at the Wilmer Eye Institute where residents are the primary providers rather than serving in a faculty member’s clinic. The resident scheduling template is increased as residents progress through their training so utilization is lower earlier in the year. In addition, it is lower than what might be expected of a faculty member. Finally, it must be noted that the no-show rate for the GES outpatient clinic averaged 22.1 % for the study period, a rate consistent with that reported recently for an academic ophthalmology resident clinic in Virginia [13]. If this rate were reduced to zero, one could estimate that the number of total visits would increase to approximately 18775; the utilization rate would increase correspondingly to 64.7 %. However, it should be mentioned that there might be negative consequences if the no-show rate were reduced to zero. For example, there might be fewer appointment slots to accept urgent walk-in (unscheduled) patients and there would be no unused appointment slots to buffer clinic flow if any particular patient required more than the usual time expected for a visit. Future studies should be performed to determine the reasons for the high no-show rate with the intent of reducing it. This would include exploring how best to adjust the scheduling template with catch-up- and urgent care-slots to provide for these exigencies.

**Table 3:** Johns Hopkins Outpatient Center - Space Utilization Summary Report FY 2014*

Department	Utilization Rate
Anesthesiology (Pre-op evaluation)	83.6 %
Medicine (Diabetes Ctr.)	91.9 %
Anesthesiology (Pain Clinic)	79.2 %
Urology	84.1 %
Radiology (Pre-interventional evaluation)	58.7 %
Neurology	102.6 %
Neurosurgery	86.6 %
Orthopedics	80.0 %
Otolaryngology	93.7 %
OB/GYN	78.1 %
Medicine (General)	80.6 %
Cardiology	63.0 %
Dermatology	136.0 %
Surgery	66.5 %
Plastic Surgery	95.4 %

*Data graciously provided by Joseph Murray and Justin Anderson with permission of Johns Hopkins Ambulatory Administration

To improve metrics such as the total number of cataract surgeries performed and the room utilization rate, it would seem that the obvious answer would be to increase patient visit volumes. However, this option may not be viable in the future because a Global Budget Revenue (GBR) contract will be enacted between hospitals such as JHH and the state of Maryland. This contract provides an annual fixed revenue to a hospital and tasks the hospital with providing care to its patients in a sufficiently cost-effective way so that the revenue covers the cost of the care [14]. The GBR contract encourages a hospital to focus on health maintenance and preventive measures, thereby reducing inpatient admissions, outpatient visits and surgical care. It is concerning that a reduction in clinic and surgical volume could actually translate into a reduction in educational opportunities for our trainees.

Perhaps a more effective opportunity for increasing cataract surgery yield might be to introduce residents to cataract surgery earlier in their training, either through virtual simulation, wet-labs or as assistant surgeons in the operating room, thereby reducing the time required for them to feel more confident and truly be more competent when assessing vision-impairing cataracts that might not be severe; the Wilmer Eye Institute is currently conducting a formal study of this approach.

Another approach for increasing cataract surgery yield (and by extension, yield for any type of surgery), would be to increase the presence of non-resident providers in the GES, such as optometrists or ophthalmic physician assistants. This model is successfully employed in private practices as a way of identifying surgical opportunities for the surgeon in numbers greater than that person might be able to do alone, and free that surgeon’s time so it can be spent in the operating room. A limitation of this approach is that residents are trainees and have a responsibility to learn many non-surgical skills prior to operating. Until further studies evaluate other academic resident ophthalmology clinics, it will be impossible to know whether the cataract surgery yield in the GES is favorable or not.

Concerning room utilization, it must be mentioned that the hospital-based view defined above is meant for situations in which the number of exam rooms is fixed but the clinic manager has discretion in how physicians are scheduled, particularly in terms of the number of providers who see patients during any given clinic session. The GES administration does not create the resident schedule, which is comprised of a series of rotations throughout the year. Rather, the GES must create a fixed schedule for the specialty clinics starting anew every July 1^st^ and secondarily accommodate the varying number of residents hosting their continuity clinic on any given day as rotations change. For a clinic such as the GES, it may be better to determine the number of patient encounters per full-time-equivalent provider or per resident clinic session. This would help clarify resident productivity and could guide decisions as to the density of the resident template. Since many residents will enter academic- and hospital-based practices, it is certainly important to educate them about metrics the hospital deems a priority (i.e., room utilization). However, it is also necessary to guide the residents about metrics that have more bearing on their own practices.

This study has some challenging limitations. Although the e-posting system seems robust, a patient can be scheduled for surgery even if the modifier we track on the electronic posting sheet is not checked by the physician planning the surgery. This would cause an under-reporting of GES surgical opportunities. Further, tracking scheduled surgical opportunities rather than accomplished surgeries may over-represent surgeries, since it is expected that some surgeries would be cancelled. Notably, the same-day cancellation for the operating room used by our patients averaged 4 % during the study period, so the effect of cancelled surgeries on the data is likely to be small. In addition, even if a surgery were cancelled, it seems reasonable to suggest that the process of evaluating and decision-making by the resident involved in examining a patient with a surgical illness is still a critical learning opportunity provided by their GES patients. Concerning the resident ACGME surgical logs, it is possible that errors of under-reporting exist, causing an overestimation of the impact of GES surgical patients on the overall resident surgical experience. Conversely, the resident logs also include cases passed to the residents by faculty. These cases may or may not be logged as ones in which the residents were the primary and therefore is a source of error.

The GES will continue to track the benchmarks described in the present study and share the data with the residents as part of their formal education in professionalism. They will be given the opportunity to share ownership of the practice management concerns of the GES in parallel with their ownership of patients they serve. It is hoped that as these ophthalmologists-in-training become familiar with benchmarking, they will be more readily able to appreciate its value in their own practices after graduation.

## Conclusions

Benchmarking for our ophthalmology resident outpatient clinic provides data that confirms the significant contribution the clinic makes toward the resident surgical experience. It also offers an avenue to introduce aspects of practice management to resident as part of their education in professionalism.

## Abbreviations

CPT: Current procedural terminology
GES: general eye services
ORMIS: operating room management information system
ACGME: accreditation council for graduate medical education
OKAP: ophthalmology knowledge assessment profile
JHH: Johns Hopkins hospital
IRB: institutional review board
ABMS: American board of medical specialties
RETF: resident education task force
DEC: division education champion
